# The cooperative regulation of miR‐221 by APE1 and AUF1 impacts p27^Kip1^ defining a miR signature relevant for cervical cancer

**DOI:** 10.1002/2211-5463.70190

**Published:** 2026-03-23

**Authors:** Matilde Clarissa Malfatti, Nicolò Gualandi, Gianluca Tell, Giulia Antoniali

**Affiliations:** ^1^ Laboratory of Molecular Biology and DNA Repair, Department of Medicine (DMED) University of Udine Italy; ^2^ Fondazione Italiana Fegato – ONLUS Liver Cancer Unit Basovizza Italy

**Keywords:** 8oxoGuo, APE1, AUF1, miR‐221, oxidation, p27^Kip1^

## Abstract

Apurinic/apyrimidinic endodeoxyribonuclease 1 (APE1) is a multifunctional protein involved in DNA repair, transcriptional regulation, and redox signaling, which significantly contributes to cancer progression and chemoresistance. Recent studies revealed a new role in RNA metabolism, specifically influencing the processing of some onco‐microRNAs (miRs). The interaction of APE1 with AU‐rich element RNA‐binding protein 1 (AUF1) is crucial in regulating RNA stability under oxidative stress conditions. Our research shows that APE1 and AUF1 bind pre‐miR‐221, preferentially once oxidized, thus affecting miR‐221 maturation and impacting gene transcription, such as the cyclin‐dependent kinase inhibitor p27^Kip1^, which is essential for tumor suppression and cell cycle regulation. Moreover, we identified a 13‐miR signature, comprising miRs regulated by both APE1 and AUF1, that exhibits strong prognostic value in cervical cancer. These findings suggest a novel potential role of both APE1 and AUF1 as therapeutic targets in cervical cancer.

## Graphical abstract

A regulatory axis involving APE1, AUF1, and miR‐221 is proposed. Pri‐miR‐221 is processed by DROSHA and DICER to generate mature miR‐221, which targets p27^Kip1^ mRNA. APE1 and AUF1 compete for pre‐miR‐221 binding. Reduced APE1/AUF1 levels impair miR‐221 biogenesis, decrease p27Kip1 mRNA degradation, and promote cell cycle progression, chemoresistance, and tumor progression. Lin, S., & Gregory, R. I. (2015). Adapted from ‘MicroRNA biogenesis pathways in cancer. Nature Reviews Cancer, 15(6), 321–333. https://doi.org/10.1038/nrc3932’. Retrieved from https://app.biorender.com/biorender‐templates.

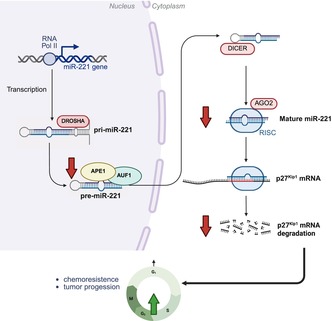

**Abbreviations**
APE1: Apurinic/apyrimidinic endodeoxyribonuclease 1
AUF1: AU‐rich element RNA‐binding protein 1
CDKN1B: cyclin‐dependent kinase inhibitor 1B
pri‐miR‐221: primary microRNA‐221, pre‐miR‐221, precursor microRNA‐221, 8oxoGuo, 8‐oxo‐7,8‐dihydroguanine

APE1 (apurinic/apyrimidinic endodeoxyribonuclease 1) is a multifunctional protein extensively studied for its roles as: (a) major endonuclease in the base excision repair (BER) pathway for maintaining genomic and mitochondrial DNA integrity, (b) transcriptional regulator, and (c) redox signaling effector [1]. Interestingly, APE1 overexpression in various cancers suggests its potential role as a key player in tumorigenesis and chemoresistance, impacting the response of cancer cells to genotoxic stress due not only to its activity in DNA repair but also in the regulation of genes involved in tumor growth [1]. Recent investigations have unveiled APE1 novel functions in RNA processing/decay, influencing gene expression by its ability to regulate RNA species and to process some onco‐microRNA (miRs) targeting genes that play a fundamental role in tumor progression [2, 3]. APE1 binds *in vitro* to RNA structures through its N‐terminal region and, as a consequence of its endoribonuclease activity, takes part in the RNA decay process [4, 5, 6, 7]. It is also known that APE1 influences gene expression due to post‐transcriptional mechanisms that involve the processing of several miRs. We demonstrated that APE1 binds to the DROSHA microprocessor, and this interaction is implicated in the maturation of pri‐miR‐221 and pri‐miR‐222, encoded in a polycistronic cluster, in response to oxidative stress in cervical cancer [6]. In this context, APE1 endoribonuclease activity on pri‐miR‐221/222 downregulates the expression of phosphatase and tensin homolog tumor suppressor (PTEN), which is a direct target of miR‐221/222, and this effect correlates with cancer progression [6]. Similarly, APE1 promotes miR‐92b maturation, affecting cervical carcinoma progression and chemosensitivity through the regulation of low‐density lipoprotein receptor (LDLR) expression [8]. Additionally, we showed that APE1 modulates RNA G‐quadruplex folding of miR‐92b itself, thus controlling its expression in cancer cells [9].

A particularly intriguing aspect of APE1 involvement in RNA metabolism is its endoribonuclease activity on abasic and oxidized ribonucleotides embedded in DNA [1, 4, 10, 11]. However, the mechanisms underlying the conversion of oxidized ribonucleotide adducts into abasic sites, as well as their potential impact on RNA structure and function, remain open questions. Oxidized miRs may also regulate cellular events by modulating corresponding effects on target genes [12], and the modulation of RNA‐ and miR‐processing and decay during genotoxic stress may be part of the executive mechanism of chemoresistance. Which proteins can specifically recognize and process oxidized miRs are unknown, and their discovery would be essential for understanding molecular mechanisms in miR quality control and developing novel specific anticancer strategies. APE1 interacts with several proteins involved in distinct cellular activities, thus regulating a broad spectrum of biological processes [5, 11, 13]. In this context, AUF1 has emerged as an important interactor of APE1, especially in relation to its activity on RNA [11]. AUF1, standing for AU‐rich element RNA‐binding protein 1, is an AU‐rich binding ribonucleoprotein (also called hnRNP D) that comprises a family of four isoforms derived from a differential alternative splicing of a common pre‐mRNA [14]. In the last years, AUF1 has been amply studied, and several functions involved in many biological processes with a strong impact on cell physiology have emerged [14, 15]. The main role of AUF1 is binding AU‐rich sequences within 3′ untranslated regions (3′UTR) of different mRNAs, thus regulating gene expression at both the transcriptional and translational levels, and coordinating functionally related pathways [15]. Recently, the role of AUF1 in miR biogenesis has also been suggested [15, 16, 17]. Indeed, AUF1 inhibits the expression of DICER, the type III ribonuclease that is required for miR maturation, by interacting with the 3′UTR of DICER mRNA itself. This interaction reduces the expression of several miRs, including miR‐122 [16], consequently affecting the regulation of their mRNA targets. Furthermore, AUF1 was found to bind miR let‐7b with high affinity and to promote the loading of let‐7b onto argonaute RISC catalytic component 2 (AGO2), thus triggering AGO2‐miR‐mediated mRNA decay [17]. Lastly, AUF1 controls miR synthesis and function by regulating their assembly into miR‐loaded RNA‐induced silencing complexes (miRISCs) and their targeting to mRNA substrates [15]. Interestingly, it has been partially demonstrated that the role of AUF1 is in the cleansing of oxidized RNA [18]. *In vitro* data have demonstrated that AUF1 preferentially binds oxidized RNA rather than unmodified RNA and is critical to the oxidative stress response [19]. Focusing on this last function, deeper studies from our laboratory have shown that AUF1 binds oxidized ribonucleotides (8‐oxo‐7,8‐dihydroguanine, 8oxoGuo) embedded in DNA [11]. In this context, we showed that AUF1 physically interacts with APE1, identifying it as a novel APE1‐interactor that stimulates APE1 endoribonuclease activity on 8oxoGuo sites in DNA [11]. Remarkably, *in‐cell* data have shown that APE1‐AUF1 interaction is modulated by exposure to different oxidative stressors and that both proteins are directly involved in the accumulation of single‐strand breaks, double‐strand breaks, and R‐loops [11]. All these premises have led us to hypothesize that APE1 and AUF1 may collaborate in the binding and processing of miRs, with a preference for the oxidized one.

In this work, we showed the relationship between APE1 and AUF1 in pre‐miR‐221 binding and biogenesis. Our *in vitro* binding data show that both APE1 and AUF1 bind to pre‐miR‐221 in its unmodified and oxidized forms, although with different efficiency. Data obtained in different cervical cancer cell lines further corroborate a possible involvement of these proteins in pri‐miR‐221 processing, as indicated by RNA immunoprecipitation (RIP) analysis and changes in the expression levels of both the primary and mature forms of miR‐221 in the presence and absence of APE1 and AUF1 proteins. This phenomenon is particularly relevant when considering the dysregulation of the cyclin‐dependent kinase inhibitor 1B (CDKN1B) gene, which encodes for p27^Kip1^, a well‐established target of miR‐221, playing a crucial role in cell proliferation [20, 21, 22]. Notably, we also demonstrated that, in addition to miR‐221, several other miRs, regulated by both APE1 and AUF1, exhibit strong prognostic value in cervical cancers, as supported by survival analysis, further reinforcing the potential of this regulatory model for the prognosis of cervical cancers.

## Materials and methods

### ‘Oligonucleotide’ annealing

Pre‐miR‐221 wild‐type (pre‐miR‐221^WT^) and pre‐miR‐221 oxidized (pre‐miR‐221^8oxoGuo^) oligonucleotides were synthesized, purified by HPLC, and lyophilized from ChemGenes Corporation (Wilmington, Massachusetts, USA) with the following sequence: 5′‐ACCUGGCAUACAAUGUAGAUUUCUGUGUUCGUUAGGCAACAGCUACAUUGUCUGCUGGGUUUC‐3′. In the pre‐miR‐221^8oxoGuo^, the guanosine in position 31st is substituted with an 8‐oxoguanosine. At the 5′ end, both oligonucleotides were labeled with the fluorophore IRDye 800 NHS Ester. Both oligonucleotides were resuspended in RNase‐ and DNase‐free H_2_O. For the annealing, a hundred picomoles were diluted in 40 μl of 10 mm Tris/HCl, pH 7.5 and 10 mm MgCl_2_, heated at 65 °C for 5 min, and then cooled down at 37 °C for 2 h in the dark.

### Protein FPLC purification and quantification

Recombinant protein purification was conducted as described previously [11, 23, 24]. After each step, the quality of the purification was verified by performing a 10% (w/v) T sodium dodecyl sulfate‐polyacrylamide gel electrophoresis (SDS/PAGE). At the end of the run, the gel was incubated in the Coomassie staining (0.05% Coomassie brilliant blue, 5% acetic acid, 50% methanol) for 10 min and then washed with the destaining buffer (25% methanol, 7% acetic acid) and then ready for the acquisition.

### Northwestern blot (NWB) analysis

NWB assay was conducted as described in [9, 13]. Briefly, 800 ng of each recombinant protein was run onto a 12% (w/v) T SDS/PAGE gel and transferred onto a nitrocellulose membrane. To allow protein denaturation, the membrane was incubated with 6 m Guanidine hydrochloride (Gdn‐HCl), freshly prepared in RNase‐free NWB buffer (10 mm HEPES pH 7.9, 50 mm NaCl, 10 mm MgCl_2_, 0.1 μm EDTA, 1 mm DTT, 50 μm ZnSO_4_, 0.1% (w/v) Tween‐20), for 10 min at room temperature (RT). After that, to allow protein renaturation, the membrane was incubated with 1 : 1 Gdn‐HCl serial dilutions six times, for 10 min at 4 °C. The membrane was then washed two times with NWB buffer and incubated with blocking buffer (5% BSA in the NWB buffer) for 1 h at RT. Then, the membrane was incubated with 2.5 pmol·μL^−1^ of annealed oligonucleotides overnight at 4 °C. The day after, the membrane was washed with the NWB buffer, ready for the acquisition.

### RNA‐protein binding assays

In the RNA‐protein binding assay, different amounts of recombinant proteins were incubated with 0.5 picomoles of each oligonucleotide in EMSA binding buffer (8 mm Hepes, 10 mm KCl, 0.4 mm EDTA, 2% glycerol) supplemented with 55 nanograms of polydIdC. In the denaturing UV‐crosslink analysis, after the incubation, the mixtures were UV‐crosslinked using a Vilber Lourmat UV‐crosslinker BLX‐245 at 0.2 J·cm^−2^, denaturized in Laemmli buffer 4× and run onto a 12% T SDS/PAGE gel. In native EMSA analysis, the samples were immediately run in a native 8% T polyacrylamide gel.

### Data acquisition and analysis

All gels and membranes were acquired with an Odyssey CLx infrared Imaging system (Li‐cor Biosciences GmbH, Germany) and analyzed by the imagestudio software.

### Cell lines and transient transfection

HeLa (RRID:CVCL_0058) and SiHa (RRID:CVCL_0032) were obtained from ATCC (Manassas, Virginia, USA) and were grown in Dulbecco's modified Eagle's medium (Euroclone, Milan, Italy) supplemented with 10% fetal bovine serum (Euroclone), 1% penicillin–streptomycin solution (100 U·mL^−1^ penicillin, 100 mg·mL streptomycin), 2 mm L‐glutamine (Euroclone) and cultured in a humidified incubator at 5% CO_2_ at 37 °C. HeLa cell clones were grown as described in [13]. The identity of the cell lines was authenticated within the past 3 years by short tandem repeat profiling. All experiments were conducted using cells confirmed to be free of mycoplasma contamination, as determined by PCR.

One day before transfection or silencing, cells were seeded in 10‐cm plates at a density of 3 × 10^6^ cells per plate. Cells were then transiently transfected with 100 pmol siRNA APE1 5′‐UACUCCAGUCGUACCAGACCU‐3′ (Dharmacon, Lafayette, Colorado, USA), ON‐TARGETplus SMART pool Human HNRNPD (L‐004079‐00‐0020; Dharmacon) or the scramble control siRNA 5′‐CCAUGAGGUCAUGGUCUGdTdT‐3′ (Dharmacon) using DharmaFECT reagent (Dharmacon). After 72 h upon transfection, cells were collected for RIP analysis and RNA quantification. For the overexpression of the APE1 and AUF1 proteins, cells were transiently transfected with 6 μg of FLAG‐tagged plasmid (please refer to [11]) using the Lipofectamine 2000 reagent (Invitrogen, Carlsbad, CA, USA), according to the manufacturer's instructions and collected 24 h after transfection.

### Luciferase assay

CDKN1B luciferase assays were performed in HeLa cells, transfected using Lipofectamine 2000 (Life Technologies, California, USA) following the manufacturer's instructions. Cells were cultured in 96‐well plates and co‐transfected with 150 ng pGL3‐p27‐3′UTR (Addgene Plasmid 20881: pGL‐p27UTR), 50 ng Renilla plasmids, and 20 nm of the APE1/AUF1 siRNA or miR‐221 mimic/negative control (NC) mimic (Dharmacon). Luciferase activity was measured 24 h after transfection using the Dual‐GLO luciferase reporter assay system (E2920; Promega, Madison, WI, USA).

### RNA immunoprecipitation (RIP)

HeLa cells were seeded in 10‐cm plates at a density of 2 × 10^6^ cells per plate. Two days before harvesting, cells were transiently silenced for the expression of APE1 and AUF1 protein (refer to the Cell lines and transient transfection paragraph). After 24 h from silencing, cells were then transiently transfected with expression plasmids for FLAG‐tagged siRNA‐resistant APE1 and AUF1. RNA immunoprecipitation was carried out as already described in [6]. The ChIP qPCR data were analyzed as previously described in [25].

### Total cell protein extract concentration and western blotting

Total cell protein extracts were prepared and quantified as already described in [11]. The protein concentration was determined by using the Bradford protein assay reagent (Bio‐Rad, Hercules, California, USA). The indicated amounts of whole cell extracts were resolved in 12% T SDS/PAGE and transferred to nitrocellulose membranes (Merck, Darmstadt, Germany). Western blot assays were performed with the following primary antibodies: anti‐APE1 (NB 100‐116, Novus Biological, Centennial, CO, USA), anti‐AUF1 (sc‐166 577; Santa Cruz Biotechnology, Dallas, TX, USA), and anti‐FLAG (F1804; Merck). Normalization was performed by using a polyclonal anti‐actin antibody (A2066; Merck). The corresponding secondary antibodies labeled with IR‐Dye (goat anti‐rabbit IgG IRDye 680 and goat anti‐mouse IgG IRDye 800) were used.

### RNA extraction and quantitative reverse transcriptase‐PCR (qRT‐PCR)

For miRs and RNAs qRT‐PCR analysis from *in vitro* cultured cell lines, RNA was isolated using the miRNeasy kit (Qiagen, Hilden, Germany), according to the manufacturer's instructions.

miR‐221 expression was validated by RT‐qPCR using TaqMan Advanced miRNA assay (Life Technologies, California, USA) [miR‐221‐3p primer (477981_mir)] following the manufacturer's instructions. Detection of successfully transcribed products was carried out using TaqMan Fast Advanced Master Mix and CFX Touch™ Real‐Time PCR System (Bio‐Rad).

For the measurement of mRNA expression and pri‐miR‐221 (Hs03303007_pri), one microgram of total RNA was reverse transcribed using the SensiFAST cDNA synthesis kit (Bioline, London, UK), according to the manufacturer's instructions. qRT‐PCR was performed with a CFX96 Real‐Time System (Bio‐Rad) using a SensiFAST SYBR No‐ROX kit (Bioline). qRT‐PCR results were calculated using the $\Delta \Delta c_{t}$ method, utilizing the expression of miR‐16‐5p (477860_mir) or GAPDH as a reference. Human primers used: CDKN1B_for 5′‐TCCGGCTAACTCTGAGGACAC‐3′ and CDKN1B_rev 5′‐TGTTTTGAGTAGAAGAATCGTCGGT‐3′; GAPDH_for 5′‐CCCTTCATTGACCTCAACTACATG‐3′ and GAPDH_rev 5′‐TGGGATTTCCATTGATGACAAGC‐3′.

### Statistical analysis

The results are presented as means ± SD, and data analysis was performed with the Prism GraphPad 7.0 software. For comparisons between two groups, unpaired and paired Student's *t*‐tests were used. In all tests, *P*‐values < 0.05 were considered statistically significant.

### Bioinformatic analysis

The prognostic significance of the identified 13 miR signature was evaluated in endocervical adenocarcinoma samples (TCGA‐CESC) from The Cancer Genome Atlas (TCGA). miR expression data for tumor samples (Illumina HiSeq platform, miRgene level, normalized, RPM) and corresponding clinical information were retrieved from the LinkedOmics portal (http://linkedomics.org/data_download/TCGA‐CESC/, last accessed: March 14, 2025). To assess the overall prognostic relevance of the miR signature, Cox regression coefficients were estimated for each miR using a multivariate Cox proportional hazards model, with miR expression levels included as covariates. For each patient, a miR score was calculated by multiplying the expression value of each miR by its corresponding Cox coefficient. The Prognostic Index (PI) was defined as the sum of these miR scores for each individual. Patients were stratified into ‘High’ and ‘Low’ groups based on optimal *P*‐value thresholds, determined using the surv_cutpoint function from the R survival package, with a minimum proportion of 0.33. Differences in overall survival (OS) between the two groups were evaluated using the log‐rank test, and survival distributions were visualized using Kaplan–Meier curves.

Correlation analyses were performed to examine the relationship between miR expression and normalized gene expression (Illumina HiSeq platform, gene‐level, normalized log_2_ RPKM; data source: http://linkedomics.org/data_download/TCGA‐CESC/, last accessed: March 14, 2025). Pearson correlation coefficients were calculated using the *cor.test* function in R. Additionally, a linear regression model was constructed to predict miR‐221 expression based on the expression levels of *APEX1* and *AUF1* genes. The model was fitted using the *lm* function in R with the formula: ‘miR‐221 ~ APEX1 + AUF1’, where miR‐221 represents the normalized miR‐221 expression and APE1 and AUF1 represent the normalized *APEX1* and *AUF1* gene expression. Component and residual plots were generated to assess model assumptions using the *crPlots* function from the *car* R package.

## Results

### APE1 and AUF1 proteins cooperatively bind pre‐miR‐221 with a preference for its oxidized form

**Fig. 1:**
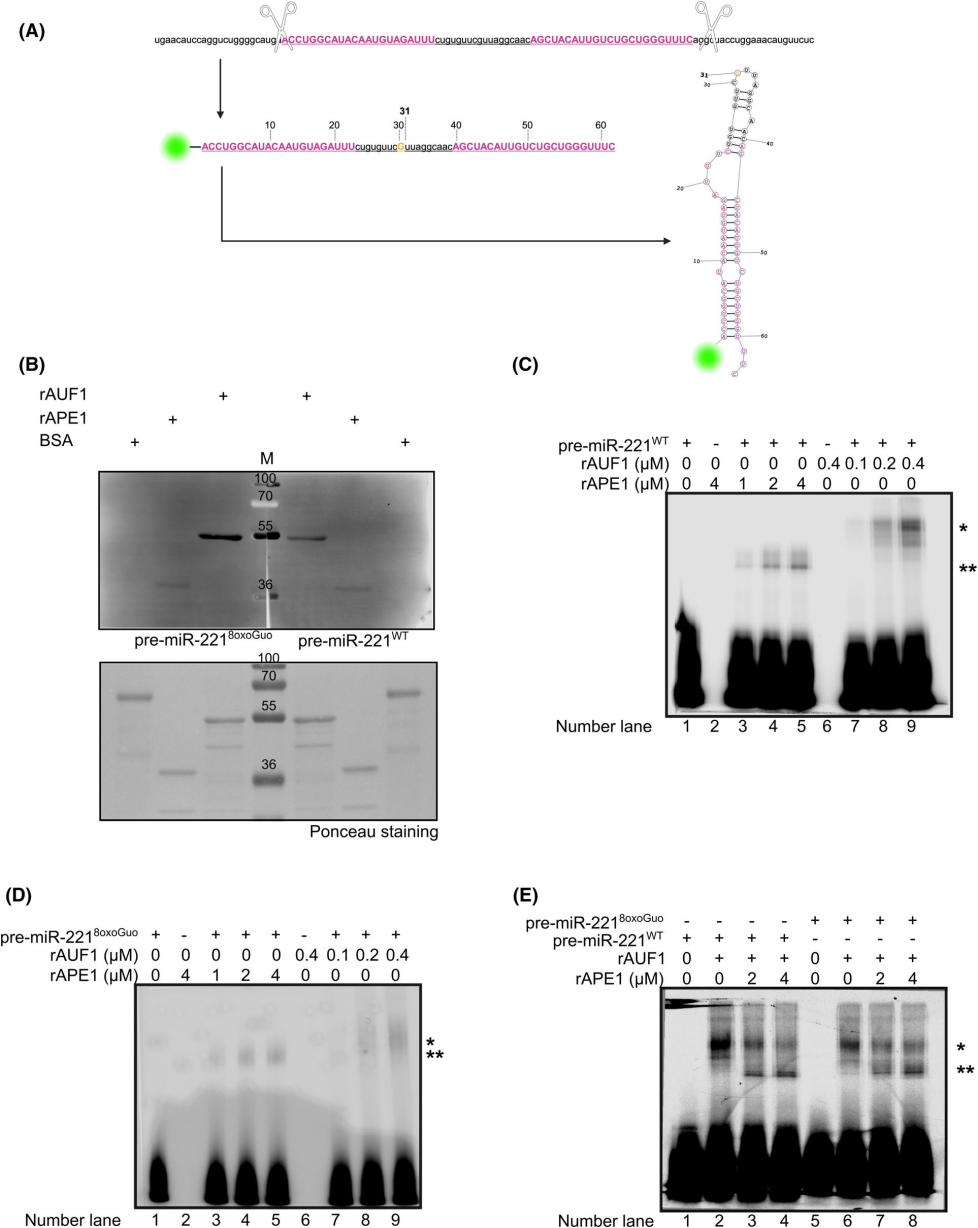
AUF1 contends with APE1 for the binding to pre‐miR‐221. (A) The 110 base‐pair (bp) pre‐miR‐221 sequence was truncated to a 63 bp sequence (underlined). This shorter sequence was labeled at the 5′ end with the IRDye NHS ester 800 fluorophore (green). At position 31, the guanine (G) was replaced with 8‐oxo‐7,8‐dihydroguanine (8oxoGuo) (yellow). The regions corresponding to the mature miR‐221‐5p (left) and miR‐221‐3p (right) forms are shown in pink. The predicted loop structure of the modified pre‐miR‐221, obtained by miRNAFold software, is also depicted. Created with Biorender.com. (B) NWB analysis for rAPE1 and rAUF1 proteins incubated with pre‐miR‐221^8oxoGuo^ (left) and pre‐miR‐221^WT^ (right). Molecular weights (m) are loaded, whose mass is expressed in kDa. (C) UV‐crosslink analysis with different amounts of rAPE1 and rAUF1 and 50 nm of pre‐miR‐221^WT^ or (D) pre‐miR‐221^8oxoGuo^, as indicated at the top of the panel. The reaction was conducted for 90 min at 4 °C. (E) UV‐crosslink analysis incubating 0.4 μm of rAUF1 with each oligonucleotide (50 nm) for 90 min at 4 °C and then adding rAPE1 (at different amounts) to the mixture for 30 min at 4 °C. Representative gel images from at least 3 independent experiments are shown. Asterisks indicate protein–probe complexes: a single asterisk (*) denotes APE1 binding, whereas a double asterisk (**) denotes AUF1 binding.

**Fig. 2:**
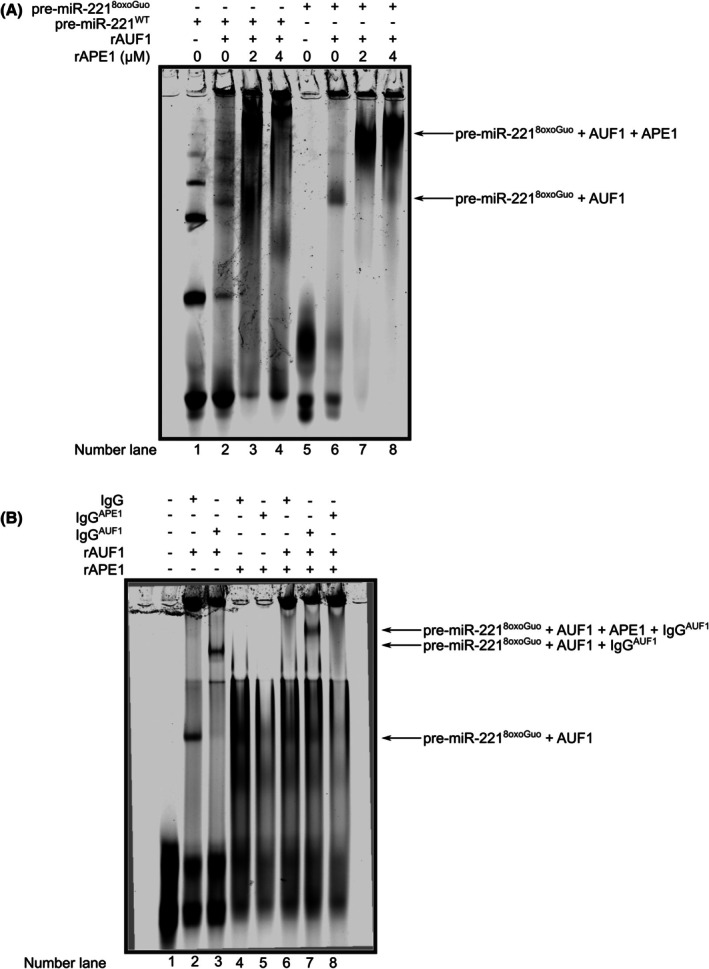
The binding of APE1 and AUF1 is specific for the oxidized pre‐miR‐221. (A) EMSA analysis was performed by mixing 4 picomoles of rAUF1 protein with 0.5 picomoles of each oligonucleotide for 90 min at 4 °C, afterwards supplemented with increasing amounts of rAPE1 as indicated on the top of the panel, for 30 min at 4 °C. On the right side of the panel, the pre‐miR‐221^8oxoGuo^ – AUF1 and the pre‐miR‐221^8oxoGuo^ – AUF1 – APE1 complexes are indicated by arrows. (B) *Supershift* EMSA analysis was conducted by incubating IgG^AUF1^ and IgG^APE1^ antibodies with 4 picomoles of rAUF1 protein and 32 picomoles of APE1, respectively, for 15 min at 4 °C. IgG was also used as a control. After that, the mixture was prepared by incubating the IgG^AUF1^ – AUF1 complex with 0.5 picomoles of pri‐miR‐221^8oxoGuo^ for 90 min at 4 °C, then supplemented with the IgG^APE1^ – rAPE1 complex for 30 min at 4 °C. On the right side of the panel, the pri‐miR‐221^8oxoGuo^ and AUF1 complex, as well as the *supershift*, are indicated by arrows. Representative gel images from at least 3 independent experiments are shown.

We previously discovered that APE1 regulates the biogenesis of miR‐221 [6] and, by interacting with AUF1, processes 8oxoGuo sites embedded in DNA [4, 11]. To investigate if both APE1 and AUF1 are also involved in the binding and processing of 8oxoGuo site in RNA, and especially in the context of the miR‐221, we decided to use, for the next *in vitro* experiments, a synthetic oligonucleotide mimicking the pre‐miR‐221 (Fig. 1A). A 110 bp pre‐miR‐221 sequence was retrieved from the *miRbase* online database. To enhance stability during *in vitro* assays, this sequence was interrupted (as indicated by the scissors symbols) to a 63 bp sequence (underlined). This shorter sequence retained the regions encoding both the mature miR‐221‐5p and miR‐221‐3p forms (pink sequences). The 63‐mer sequence was then synthesized and 5′‐end labeled with the IRDye NHS Ester 800 dye. Structural prediction analysis, obtained by miRNAFold software [26, 27], confirmed that the shortened sequence maintained the characteristic loop structure of pre‐miR‐221. To investigate the effect of oxidation, two versions of the pre‐miR‐221 oligonucleotide were used: a wild‐type version (pre‐miR‐221^WT^) and an 8‐oxo‐7,8‐dihydroguanine (8oxoGuo)‐containing version (pre‐miR‐221^8oxoGuo^). In the 8oxoGuo version, the guanine (G) at the 31st nucleotide position (highlighted in yellow) was replaced by 8oxoGuo. By using both oligonucleotides, we first performed an NWB assay with APE1 and AUF1 purified recombinant proteins (rAPE1 and rAUF1, respectively) (Fig. 1B). Briefly, each recombinant protein was run in a denaturing SDS/PAGE gel and blotted onto nitrocellulose, including bovine serum albumin (BSA) as a negative control. Then, blotted proteins underwent renaturation with serial dilutions of GdnHCl, followed by incubation with both labeled oligonucleotides, separately. As shown in Fig. 1B, although both proteins bound the two oligonucleotides, we observed a preference of APE1 for binding the oligonucleotide compared to AUF1. These data were further confirmed by UV‐crosslinking assay in which rAPE1 and rAUF1 proteins were incubated with both pre‐miR‐221 oligonucleotides under native conditions, followed by UV‐crosslinking and SDS/PAGE gel analysis (Fig. 1C,D). Interestingly, both APE1 and AUF1 bound pre‐miR‐221^WT^ (Fig. 1C) and pre‐miR‐221^8oxoGuo^ (Fig. 1D), forming distinct and well‐detectable higher‐order molecular complexes in a dose‐dependent manner (lanes 3‐4‐5 for rAPE1 and 7‐8‐9 for rAUF1 indicated by a single and a double asterisk, respectively). Finally, to check whether APE1 and AUF1 may compete for the binding to pre‐miR‐221, we performed a UV‐crosslinking analysis by pre‐incubating rAUF1 with each oligonucleotide, followed by the addition of rAPE1 (Fig. 1E). For both oligonucleotides, we observed that, in the presence of rAPE1, the rAUF1‐pre‐miR‐221 complex, indicated by a single asterisk (lanes 2 and 6), was less shifted (lanes 3–4 for pre‐miR‐221^WT^ and 7–8 for pre‐miR221^8oxoGuo^), indicated by a double asterisk (Fig. 1E). The latest finding strongly suggests that, although both rAPE1 and rAUF1 proteins bound to both WT and oxidized pre‐miR‐221, forming a macro‐crosslinked complex, there was a competition for the binding to both oligonucleotides. Subsequently, when we performed the binding analyses under native conditions, through EMSA (Fig. 2A), we observed that the incubation of rAUF1 resulted in a clear shift of pre‐miR‐221^8oxoGuo^ (lane 6), while no stable complex was detectable with pre‐miR‐221^WT^ (lane 2), already observable in lane 1, in which any protein is missing. Furthermore, in contrast to the results obtained in the UV‐crosslinking assay, upon rAPE1 addition to the reaction, we observed a supershift with pre‐miR‐221^8oxoGuo^ (lanes 7 and 8). This suggests that APE1 and AUF1 may bind together to pre‐miR‐221^8oxoGuo^, forming a macromolecular complex rather than competing for binding. This is particularly clear for the oxidized form of pre‐miR‐221, while no clear data were obtained in the case of pre‐miR‐221^WT^ (lanes 3 and 4). Lastly, by focusing our attention on pre‐miR‐221^8oxoGuo^, we performed a supershift EMSA assay by using antibodies directed *against* rAPE1 and rAUF1 (Fig. 2B). We observed a supershift of the rAUF1‐pre‐miR221^8oxoGuo^ complex (lane 2) in the presence of the antibody directed *against* AUF1 (IgG^AUF1^ – lane 3). Interestingly, the complex rAPE1‐rAUF1‐pre‐miR221^8oxoGuo^ (lane 6) was shifted in the presence of the IgG^AUF1^ (lane 7) and even more in the presence of the antibody‐directed *versus* APE1 (IgG^APE1^ – lane 8). Overall, these data support the hypothesis that APE1 and AUF1 may collaborate in the binding of the pre‐miR‐221 with a preference for the oxidized one.

### APE1 and AUF1 regulate p27^Kip1^ expression through miR‐221

**Fig. 3:**
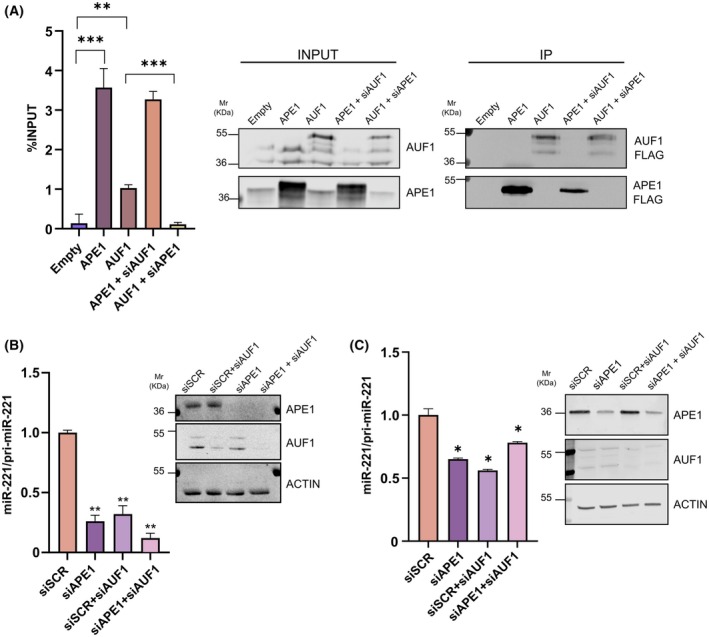
Impact of APE1 and AUF1 in miR‐221 biogenesis. (A) RIP analysis of pri‐miR‐221 bound by APE1 and AUF1. RT‐qPCR was performed on HeLa cells transiently transfected with empty plasmid (EMPTY), APE1‐FLAG‐tagged protein (APE1), AUF1‐FLAG‐tagged protein (AUF1) and simultaneous transfection and silencing of APE1 and AUF1, respectively (AUF1 + siAPE1, APE1 + siAUF1). Fold enrichment of pri‐miR‐221 immunoprecipitation was calculated as described in [25]. Western blotting analysis was performed on total cell extracts (INPUT) and FLAG‐immunoprecipitated material (IP) with specific antibodies. (B, C) Mature miR to pri‐miR ratios in cells silenced for APE1 and AUF1 expression. miRNA was extracted from HeLa cell clones (B) stably transfected with a scramble siRNA control (siSCR), with APE1 siRNA (siAPE1), transiently silenced for AUF1 protein (siAUF1) and silenced for both APE1 and AUF1 (siAPE1 + siAUF1) or from SiHa cells transiently transfected with siRNA (C). Mature miR‐221 was measured by qRT‐PCR analysis, normalized to miR‐16, and expressed relative to GAPDH‐normalized pri‐miR‐221. On the right, western blotting analysis showing the silencing of APE1 and AUF1 proteins. For all panels: mean ± SD, *n* = 3, * *P* < 0.05, ** *P* < 0.01, *** *P* < 0.001, Student *t*‐test.

To assess the biological relevance of the potential cooperation of APE1 and AUF1 in the processing of miR‐221 in cancer cells, we initially validated our *in vitro* binding observations by performing RIP analysis. HeLa cells were transiently transfected with plasmids encoding FLAG‐tagged APE1 (APE1) and AUF1 (AUF1) proteins, and then, the immunoprecipitated RNA was analyzed by qRT‐PCR to quantify the levels of pri‐miR‐221 bound and compared to the Empty vector (Fig. 3A). The complete pri‐miR sequence was inspected as this would provide comprehensive insights into the regulation of miR biogenesis by APE1 and AUF1. Both proteins showed proficient binding to pri‐miR‐221. More interestingly, when we analyzed RIP in the context of APE1 and AUF1 reciprocal silencing to better circumstantiate their mutual contributions, we observed a significant decrease in AUF1 binding upon APE1 silencing, while the APE1 binding was not significantly affected by AUF1 silencing. This result confirmed the previous crosslinking data (Fig. 1E) of a possible competitive relationship between the two proteins in favor of APE1 and also supported our *in vitro* characterization for a collaborative binding to pri‐miR‐221.

Therefore, considering the potential function of APE1 and AUF1 proteins in regulating miR‐221 processing, we investigated their role in miR‐221 processing efficacy by checking the ratio of miR‐221 precursor and mature form expression upon silencing each of the two proteins alone or in combination in HeLa cells clones (Fig. 3B). Silencing of APE1 and AUF1 resulted in an imbalanced expression of miR‐221 as reported by the ratio of mature/immature form and as a consequence of the increased expression of the precursor form. In particular, the concomitant silencing of the two proteins resulted in an augmented impairment of the expression of miR‐221. The relevance of this regulatory mechanism is further underscored by its replication in SiHa cell lines, another widely used cervical cellular model, in which AUF1 and APE1 silencing produced comparable effects on miR‐221 biogenesis (Fig. 3C).

**Fig. 4:**
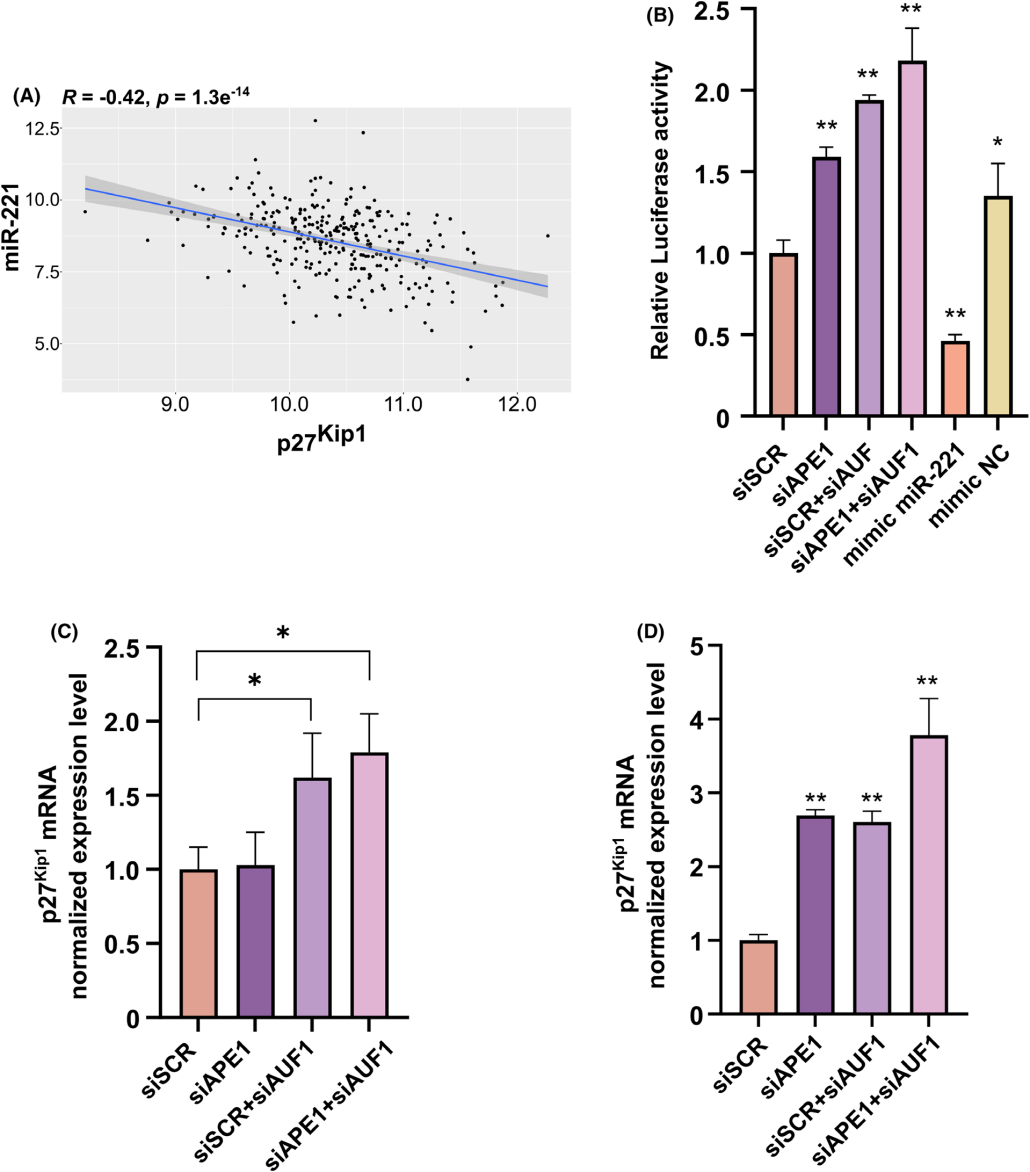
Influence of APE1 and AUF1 on the control of p27^Kip1^ levels. (A) Correlation between p27^kip1^ and miR‐221 expression. The *x*‐axis shows the normalized p27^kip1^, while the *y*‐axis shows the normalized miR‐221 expression. The Pearson correlation coefficient (*R*) and the *P*‐value are displayed at the top of the plot. (B) Luciferase reporter assay performed by co‐transfecting HeLa cells transiently silenced for APE1 and AUF1 with a Firefly luciferase reporter containing the p27 3′UTR and a control Renilla luciferase plasmid. MiR mimic for miR‐221 was used as a positive control, along with its corresponding negative control (mimic NC). Firefly luciferase activity was normalized to the Renilla signal, and data are expressed with respect to siSCR. (C, D) qRT‐PCR analysis of p27^Kip1^ mRNA level as miR‐221 target in HeLa cell clones (C) and SiHa cells (D). Data are normalized with respect to GAPDH levels. For all panels: mean ± SD, *n* = 3, *P < 0.05, **P < 0.01, ***P < 0.001, Student *t*‐test.

In order to detect the existence of any functional relevance of the miR‐221 impairment, we evaluated the expression of a known target of miR‐221 that is CDKN1B/p27^Kip1^ [20, 21, 22]. Interestingly, we observed a significant negative correlation (*R* = −0.44, *P*‐value = 1.3 × 10^−14^) between miR‐221 and CDKN1B/p27^Kip1^ expression in TCGA‐CESC (Fig. 4A).

To further confirm that reduced miR‐221 activity affects p27^Kip1^ regulation, we next performed a luciferase reporter assay using the 3′UTR of p27^Kip1^ (Fig. 4B). Silencing of APE1 and/or AUF1 resulted in a significant derepression of luciferase activity, consistent with impaired miR‐221‐mediated repression as observed when co‐transfecting the miR‐221 mimic. In line with these findings, when we examined p27^Kip1^ expression in HeLa cells (Fig. 4C) or in SiHa cells (Fig. 4D) upon silencing of APE1 and AUF1, we observed an increased expression, which inversely correlated with the decrease of miR‐221/pri‐miR‐221 expression in the same context.

These findings support the existence of an APE1/AUF1/miR‐221 regulatory axis in cervical cancers, which ultimately may affect p27^Kip1^ tumor suppressor expression.

### APE1‐ and AUF1‐regulated miR signature shows a prognostic power in cervical cancers

**Fig. 5:**
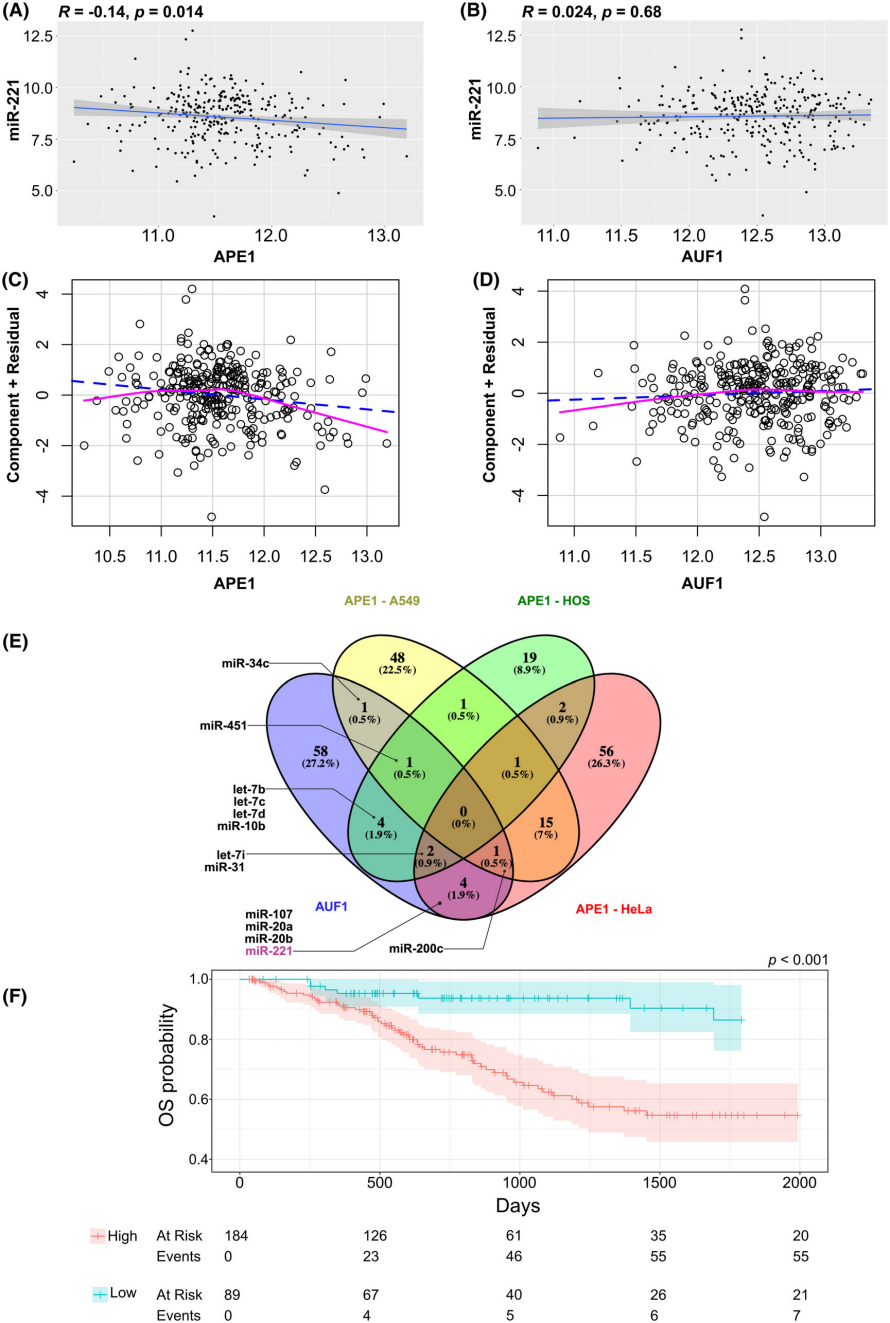
Prognostic value of 13 miR signature in association with APE1 and AUF1. (A) Correlation between APE1 and miR‐221 expression. The *x*‐axis shows the normalized APE1 levels, while the *y*‐axis shows the normalized miR‐221 expression. The Pearson correlation coefficient (*R*) and the *P*‐value are displayed at the top of the plot. (B) Correlation between AUF1 and miR‐221 expression. The *x*‐axis shows the normalized AUF1, while the *y*‐axis shows the normalized miR‐221 expression. The Pearson correlation coefficient (*R*) and the *P*‐value are displayed at the top of the plot. (C) Component and residuals plot for the linear model predicting miR‐221 expression from APE1 and AUF1 expression. The *x*‐axis represents the expression levels of APE1, while the *y*‐axis shows the residuals of the predicted miR‐221 expression. This plot assesses the fit of the linear regression model by displaying the differences between the observed and predicted values of miR‐221 expression. (D) Component and residuals plot for the linear model predicting miR‐221 expression from APE1 and AUF1 expression. The *x*‐axis represents the expression levels of AUF1, while the *y*‐axis shows the residuals of the predicted miR‐221 expression. This plot assesses the fit of the linear regression model by displaying the differences between the observed and predicted values of miR‐221 expression. (E) A Venn diagram was generated by using venny 2.1 online software [28]. Common miR between AUF1′ and APE1′ lists are depicted. (F) Kaplan–Meier plots of the TCGA‐CESC dataset illustrate the differences in overall survival (OS) between subjects classified as ‘High’ and ‘Low’ groups, stratified according to the Prognostic Index (PI) derived from the 13‐miRNA signature. Results are displayed up to 2000 days, as the sample size beyond this point is insufficient for reliable statistical inference.

To confirm our data obtained *in vitro* and in immortalized cells regarding the biological role of both APE1 and AUF1 in the miR‐221 regulation, we next examined the possible correlation existing between miR‐221 expression and the expression levels of *APEX1* and *AUF1* genes. We observed a statistically significant negative correlation between APE1 and miR‐221 expression (*R* = −0.14, *P* = 0.014), whereas no significant correlation was detected between AUF1 and miR‐221 expression (Fig. 5A,B). Furthermore, a linear regression model constructed to predict miR‐221 expression based on APE1 and AUF1 levels confirmed that APE1 exhibited substantial predictive power, while AUF1 showed little to no association with miR‐221 expression (Fig. 5C,D). In addition to miR‐221, several other miRs appear to be potentially regulated by both APE1 and AUF1 proteins [2, 6, 28, 29]. With the aim of identifying miRs commonly regulated by both APE1 and AUF1 proteins, we cross‐referenced miRs regulated by APE1 derived from RNAseq analysis performed in our laboratory in HeLa [6] and A549 cells [2], together with those obtained in HOS cells [28], with those reported to be regulated by AUF1 [29]. This integrative analysis revealed that, beyond miR‐221, several additional miRs are co‐regulated by both proteins including miR‐34c, miR‐451, miR‐200c, miR‐20a, miR‐20b, miR‐107, miR‐31, miR‐10b, let‐7b, let‐7c, let‐7d, let‐7i (Fig. 5E). Interestingly, the selected 13 miRs signature demonstrated a significant prognostic power in the TCGA‐CESC dataset, suggesting that the expression levels of these miRs are highly predictive of cervical cancer patient outcomes. Specifically, the expression of the 13‐miR signature was significantly associated with distinct overall survival (OS) probabilities, with patients classified as ‘Low’ expression showing an increase OS probability over 2000 days, further supporting its potential prognostic value in the TCGA‐CESC dataset (Fig. 5F, *P* = 0.0001, Low group = 184, High group = 89, Hazard ratio Low/High = 0.31).

## Discussion

Several RNA‐binding proteins (RBPs), including AUF1, play critical roles in various types of cancer, and their aberrant expression in cancer cells makes them an attractive therapeutic target for cancer treatment [30]. Oxidative stress contributes to tumorigenesis by altering gene expression by affecting DNA [31], but also RNA, if oxidized, can affect cell physiology [32]. For example, the conversion of guanosine into 8‐oxoGuo can change RNA–RNA interactions by affecting the canonical Watson‐Crick base pairing, but its regulatory roles remain elusive. Recently, it has been discovered that a widespread 8‐oxoguanosine modification in onco‐miRs occurs in the seed regions, close to the 5′ end (positions 2–8) with clustered sequence patterns [12], and is clinically associated with patients affected by lower‐grade gliomas and liver hepatocellular carcinoma, thus suggesting the existence of an epitranscriptional 8‐oxoguanosine regulation of miR functions [12]. Mechanistic insights into the functional role and processing of oxidized miRs are still missing.

APE1 is overexpressed in the nucleus and cytoplasm of many tumor types and contributes to the expression of chemoresistance genes, also through its functions in RNA metabolism, including miRs [1].

In this context, using HeLa cells under genotoxic stress conditions, we have demonstrated that nuclear APE1 favors the processing and stability of the miR precursors through the association with DROSHA. This phenomenon impacts the miR‐221/222 axis and modulates the expression of the tumor suppressor PTEN [6]. We previously showed that, in lung cancer, a signature of specific miRs (i.e., miR‐1246, miR‐4488, miR‐24, miR‐183, miR‐660, miR‐130b, miR‐543, miR‐200c, miR‐376c, miR‐218, miR‐146a, miR‐92b, and miR‐33a) is strongly correlated with APE1 expression and is involved in the regulation of DICER expression and as well as in EMT in NSCLC cancer cell lines [2]. This evidence substantiates the notion that the APE1‐oncomiRs axis plays a pivotal role in cancer cell response to genotoxic treatments, elucidating the involvement of APE1 in chemoresistance through post‐transcriptional mechanisms.

Furthermore, APE1 roles are specifically modulated by its interaction with different protein partners involved in RNA metabolism and miRs sorting (e.g., NPM1, hnRNPA2/B1, AUF1, FUS, SFPQ) [33, 34, 35]. Under genotoxic stress conditions, we established that APE1 is involved in the decay of cisplatin‐damaged RNA in association with nuclear exosome proteins (i.e., MTR4) [13] and in the processing of 8oxoGuo embedded in DNA, in association with proteins recognizing oxidized RNA, including AUF1 [11].

Therefore, considering the possibility that APE1 may play a role in the processing/decay of oxidized miRs, we here deepen the molecular mechanism of action of APE1 towards the oxidized pre‐miR‐221 in association with its known protein partner, AUF1. AUF1 is of particular interest because of its recent implication in miR stability and decay [16, 17]. Beyond its role in mRNA regulation, recent studies indicated that AUF1 may regulate miR biogenesis and stability through various mechanisms: (a) direct interaction with mature and precursor miRs [29]; (b) modulation of RISC complex stability [36]; (c) control of specific miRs under stress responses [37]; and (d) competitive interaction with other RBPs for the binding sites on mRNA, thereby affecting miRs availability [38]. Considering all these aspects, we envision that APE1 and AUF1 could be involved together in the binding and processing of some miRs, particularly under oxidative stress conditions.

Taking advantage of a probe mimicking the precursor form of miR‐221 (pre‐miR‐221) in both normal (pre‐miR‐221^WT^) and oxidized (pre‐miR‐221^8oxoGuo^) versions, we highlighted that both APE1 and AUF1 proteins are capable of binding the pre‐miR‐221^8oxoGuo^. In particular, when looking at the UV‐crosslinking assay data obtained, in which the binding was stabilized, we observed a competition between the two proteins, probably due to a possible binding overlap between the two proteins. Interestingly, through nondenaturing gel, we confirmed that presumably APE1 and AUF1 may form a supramolecular RNA‐protein complex with the pre‐miR‐221^8oxoGuo^ thus suggesting that the binding equilibrium may be in favor of the oxidized form. This could be supported by the speculation that the damaged miR is degraded in the cell. Furthermore, it was interesting to note that data obtained in HeLa cells support the potential role of APE1 and AUF1 in the miR‐221 precursors binding and in its processing, thus impacting its biogenesis. This involvement was supported by the RIP analysis data and by the observed changes in the expression levels of both the precursor and mature forms, depending on the presence or absence of these proteins. This mechanism is especially relevant in cancer cells, given that miR‐221 targets CDKN1B/p27^Kip1^, a cyclin‐dependent kinase inhibitor crucial for regulating cell proliferation and involved in chemoresistance.

Given the observed overexpression of both APE1 and AUF1 in various tumors [11] and understanding their roles in miRs processing within cancer cells, this proof‐of‐concept study suggests that selectively targeting their function could be a valuable strategy for sensitizing resistant cells to chemotherapy. Indeed, by performing a cross‐analysis on datasets of miRs individually regulated by AUF1 and APE1, we identified a shared subset of 13 miRs, including miR‐34c, miR‐451, let‐7b, let‐7c, let‐7d, let‐7i, miR‐10b, miR‐31, miR‐107, miR‐20a, miR‐20b, miR‐200c, in addition to miR‐221. Survival analysis using the TCGA‐CESC dataset demonstrated that this 13‐miRNA signature has significant prognostic power in cervical cancer, indicating that co‐regulation by APE1 and AUF1 may define a clinically relevant profile associated with patient outcomes.

Our work represents a first mechanistic attempt towards answering whether these two proteins can be involved in the recognition and decay of oxidatively damaged miRs. Further work needs to be done along this line. Although the quantitative measurement of endogenous oxidized miRs still remains technically challenging and was not feasible in this study, we are actively working to overcome this limitation. Answering this question will be essential for understanding how AUF1 and APE1 affect miRs' stability and function under oxidative stress conditions and could have significant implications for therapeutic interventions in diseases involving oxidative RNA damage and inflammation.

## Conflict of interest

The authors declare no conflict of interest.

## Author contributions

GA together with MCM, GT conceived and designed the study and supervised the experiments. MCM performed EMSA, crosslink and NWB experiments. GA performed RIP analysis, luciferase reporter assay and qPCR. NG performed bioinformatic analysis. GA and MCM analyzed and interpreted all data, and mainly wrote the manuscript. GT provided financial support and provided critical comments and suggestions and contributed to the interpretation of the results. All authors critically read and approved the final version of the manuscript.

## Data Availability

The datasets used and/or analyzed in this study are available from the corresponding author upon request.
